# Personal Accounts of Young-Onset Colorectal Cancer Organized as Patient-Reported Data: Protocol for a Mixed Methods Study

**DOI:** 10.2196/25056

**Published:** 2021-02-26

**Authors:** Klay Lamprell, Diana Fajardo Pulido, Yvonne Tran, Bróna Nic Giolla Easpaig, Winston Liauw, Gaston Arnolda, Jeffrey Braithwaite

**Affiliations:** 1 Australian Institute of Health Innovation Macquarie University Sydney Australia; 2 St. George Cancer Care Centre St. George Hospital Sydney Australia

**Keywords:** colorectal cancer, PROMs, young-onset cancer, cancer, patient reported outcome

## Abstract

**Background:**

Young-onset colorectal cancer is a contemporary issue in need of substantial research input. The incidence of colorectal cancer in adults younger than 50 years is rising in contrast to the decreasing incidence of this cancer in older adults. People with young-onset colorectal cancer may be at that stage of life in which they are establishing their careers, building relationships with long-term partners, raising children, and assembling a financial base for the future. A qualitative study designed to facilitate triangulation with extant quantitative patient-reported data would contribute the first comprehensive resource for understanding how this distinct patient population experiences health services and the outcomes of care throughout the patient pathway.

**Objective:**

The aim of this study was to undertake a mixed-methods study of qualitative patient-reported data on young-onset colorectal cancer experiences and outcomes.

**Methods:**

This is a study of web-based unsolicited patient stories recounting experiences of health services and clinical outcomes related to young-onset colorectal cancer. Personal Recollections Organized as Data (PROD) is a novel methodology for understanding patients’ health experiences in order to improve care. PROD pivots qualitative data collection and analysis around the validated domains and dimensions measured in patient-reported outcome and patient-reported experience questionnaires. PROD involves 4 processes: (1) classifying attributes of the contributing patients, their disease states, their routes to diagnosis, and the clinical features of their treatment and posttreatment; (2) coding texts into the patient-reported experience and patient-reported outcome domains and dimensions, defined a priori, according to phases of the patient pathway; (3) thematic analysis of content within and across each domain; and (4) quantitative text analysis of the narrative content.

**Results:**

Relevant patient stories have been identified, and permission has been obtained for use of the texts in primary research. The approval for this study was granted by the Macquarie University Human Research Ethics Committee in June 2020. The analytical framework was established in September 2020, and data collection commenced in October 2020. We will complete the analysis in March 2021 and we aim to publish the results in mid-2021.

**Conclusions:**

The findings of this study will identify areas for improvement in the PROD methodology and inform the development of a large-scale study of young-onset colorectal cancer patient narratives. We believe that this will be the first qualitative study to identify and describe the patient pathway from symptom self-identification to help-seeking through to diagnosis, treatment, and to survivorship or palliation for people with young-onset colorectal cancer.

**International Registered Report Identifier (IRRID):**

DERR1-10.2196/25056

## Introduction

Routine systematic collection of patient-reported outcome (PRO) and patient-reported experience (PRE) data is of considerable interest to health systems worldwide and is the subject of ongoing investment [1,2]. Validated instruments—most often in the form of standardized questionnaires—are regularly used to measure patients’ perspectives on the quality of health services and personal outcomes of clinical management care. These data are considered foundational in understanding the effects of health care on patients’ daily lives [3] and for making improvements in health care delivery [4,5]. Mixed-methods approaches [6] are increasingly becoming common in the collection of PRO and PRE data. Measurement instruments are sometimes supplemented with open-ended, free-text questions [7] to capture nuanced and idiosyncratic perspectives [5,8-10]. This descriptive material [7,11] has been shown to contextualize responses to closed questions [12] to provide more detail about the relational aspects of patients’ experiences [11] and to be more specific about the aspects of care that can be improved to promote better outcomes [11-13].

Qualitative researchers investigating patients’ experiences of care and perspectives on outcomes may have opportunities to facilitate mixed-methods approaches [6] for the collection of patient-reported data. In this paper, we present a methodology for producing qualitative data that effectively triangulates [6,14,15] with quantitative colorectal PRO and PRE data [16-19]. The methodology, which we call as Personal Recollections Organized as Data (PROD), pivots data collection and analysis around the validated domains and dimensions measured by PRO and PRE instruments [20-23]. The aim is to facilitate synthesis of patient-reported evidence across research projects. To our knowledge, this is a novel approach to qualitative patient experience data collection.

PROD draws on the “framework method” [24,25], in which free text or narrative data are organized into classifications that have been determined a priori and utilizes thematic/inductive techniques to facilitate the interpretation of emergent PRE and PRO topics [16-19], including quantitative text mining techniques, which are a resource-efficient means of identifying patterns and modelling relationships between topics [12,24,25].

The PROD method will be used to investigate the perspectives of people with young-onset colorectal cancer. The increasing incidence of colorectal cancer in people younger than 50 years has been described as an alarming phenomenon [26] within the wider population of patients with colorectal cancer [27-32]. The incidence of young-onset colorectal cancer has risen by up to 2% per year worldwide while that of colorectal cancer in older adults is declining by up to 3% per year [26,31,33,34]. Dietary and lifestyle changes framed by shifts in global food chains have been proposed as causes for the rise in young-onset colorectal cancer [35]. Additionally, colorectal cancer awareness campaigns and screening programs are directed at people aged 50 years and older [35]. Patients with colorectal cancer who are younger than 50 years are twice as likely as older patients to experience missed diagnostic opportunities by physicians [36], significantly more likely to be diagnosed at an advanced stage of the disease [30,32], have a greater likelihood of aggressive therapeutic management [32], and will commonly have poorer quality of life outcomes [13,37-39].

We have knowledge of this patient population from age-stratified data of the wider colorectal cancer population; however, there has been limited attention on patients with young-onset colorectal cancer as a specific patient community. Patients younger than 50 years are at that stage of life in which they are establishing careers, building relationships with long-term partners, raising children, and assembling a financial base for the future. Their perspectives on their experiences of health services and outcomes of care may be different from those of older patients with colorectal cancer.

Our study aims to address the gap in qualitative patient-reported data on young-onset colorectal cancer by investigating the personal accounts published online by these patients. Web-based autobiographical accounts of health care experiences and outcomes are emerging sources of qualitative patient-reported data on disease-specific and condition-specific patient experience [40-42]. The accounts we will access are extant texts [20,43] in contrast to interactive forms of web-based self-narration in blogs and social media, which have been investigated elsewhere [44,45]. These unsolicited narratives, not produced in response to a research inquiry [20], provide rich detail on the health care experiences and issues that matter to these patients [42].

Patient narratives commonly describe the entire health care journey—from initial help-seeking to current survivor or palliative care status [40]—from the patients’ points of view [40]. They feature highly personal perspectives on the performance of health services and physical, emotional, and social outcomes of medical management across the trajectory of care [42]. As sources of patient-reported data, these narratives offer a counterpoint to data produced from cross-sectional surveys. They provide significantly more descriptive data than those that can be derived from supplementary free-text questions in PRE and PRO questionnaires. Given that qualitative research by participant interview can be a labor-intensive and time-intensive process, there is an advantage also in the accessibility of patients’ unsolicited narratives with respect to ethical considerations [46]. The PROD methodology, with its clear thematization of coding around existing PRE/PRO dimensions, offers access to rich, longitudinally framed, patient-reported data.

## Methods

### Design Methodology

**Figure 1:**
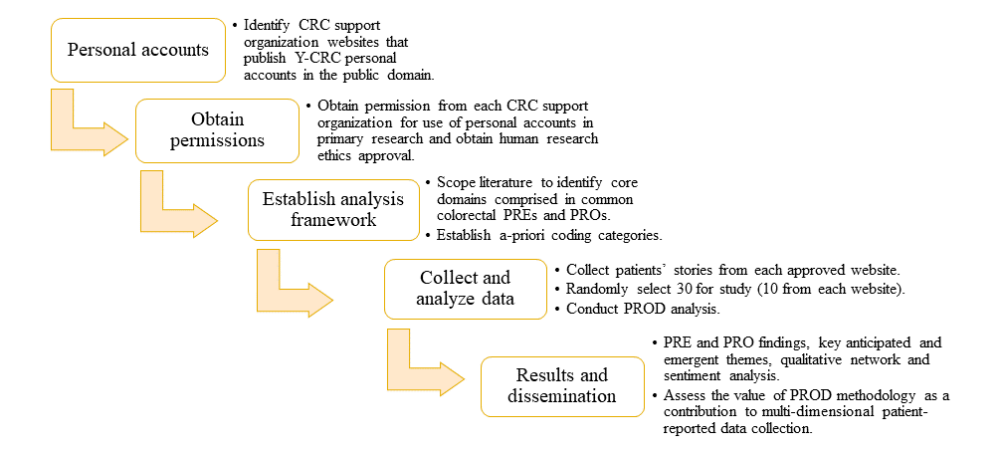
Flowchart of the study design. CRC: colorectal cancer; Y-CRC: young-onset colorectal cancer; PRE: patient-reported experience; PRO: patient-reported outcome; PROD: personal recollections organized as data.

A flowchart of the study design is depicted in Figure 1. This is a study of personal patient stories published on websites hosted by 3 established colorectal disease support organizations: Bowel Cancer Australia, Bowel Cancer UK, and Bowel Cancer NZ. These countries were chosen as they are all English-speaking and have universal health care access. This project will access the public domain sections of these websites in which people post accounts of their experiences under banners such as “real life stories” or “your stories.”

### Ethical Considerations

There is no established ethical stance relating specifically to research involving unsolicited web-based narratives. We have obtained permission from each of the organizations to analyze these personal accounts and to use deidentified excerpts and quotes in reports of findings from the study. The organizations that host the websites have agreements with individual patient contributors regarding the use of their information and narrative material. These contributors are not direct participants of our study. However, the study of unsolicited autobiographical narratives is a unique research space with particular ethical issues relating to recruitment [47]. To establish the ethical position of this study, we refer to the Australian National Statement on Ethical Conduct in Human Research (2007-Updated 2018) [48], which indicates that privacy concerns arise when the proposed access to, or use of, the data or information does not match the expectations of the individuals from whom this data or information was obtained or to whom it relates. Therefore, we were granted ethical and scientific approval for this project from the Macquarie University Human Research Ethics Committee (MQ HREC Reference No:52020666115757). In publishing their personal accounts on the selected colorectal support organization websites, these contributors agreed that their stories would be made available for public access and used to raise awareness of young-onset colorectal cancer. This study meets the expectations of the contributors. Moreover, this study does not place burdens of active research participation on these potentially vulnerable contributors [49,50]. Additionally, unsolicited accounts enact the values of patient-reported data.

### Recruitment

We defined inclusion and exclusion criteria to identify the types of personal accounts published on these sites that would be relevant to the study’s aims. We will include personal accounts that are written by people diagnosed with colorectal cancer (self-reported disease state, including but not limited to cancer of the colon, cancer of the rectosigmoid junction, and cancer of the rectum) [13]; before their 50th birthday; published in the public domain spaces of websites hosted by the 3 prominent colorectal disease support organizations, under agreement for the public dissemination and republication of the material; written by people aged 18 years or older at the time of submitting their personal accounts for publication on the website; and autobiographical, first-person accounts of experiences and outcomes relating to care for colorectal cancer. We will exclude personal accounts from the study if they solely comprise feedback on, or criticism of, a named institution or clinician or substantially describe someone else’s experiences and outcomes relating to care for young-onset colorectal cancer. We are not including serialized narrative material published as ongoing weblogs or blogs. We will take a random sample of 30 personal accounts from the eligible selection of personal stories using the Microsoft Excel (2011) random function, comprising 10 samples from the patient stories published on each of the 3 websites.

### Data Extraction and Analysis

**Figure 2:**
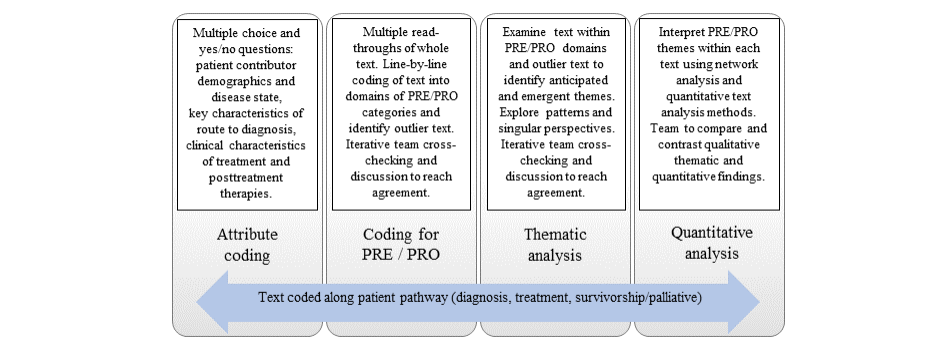
Overview of the framework for analysis. PRE: patient-reported experience; PRO: patient-reported outcome.

Narrative accounts will be downloaded from websites and collected and analyzed using the qualitative analysis software NVivo 12 Plus (QSR International) [51]. To avoid identification of individuals, each story will be deidentified and assigned a unique identifier code. Our framework method for qualitative analysis [24] identifies a priori what features to account for in our research reporting [16]. We detail our process for establishing the analytical framework in the following section on PRE and PRO domain coding. The PROD approach involves 4 key steps in creating a new structure for the data, as shown in Figure 2: (1) classifying attributes of the contributors, their disease states, their routes to diagnosis, and the clinical features of their treatment and posttreatment; (2) coding each line of each narrative into PRE and PRO categories and domains according to phases of the patient pathway; (3) thematic analysis of content within each domain; and (4) quantitative analysis of the narrative content.

Manual coding and analysis will be undertaken by the process of line-by-line attention to the content in a series of iterative readings. Consistent with the principles of qualitative research, each step of the data extraction and analytical process will be undertaken by at least two researchers [18], as qualitative work with narrative data is interpretive, even when coding to a framework of categories and domains established a priori.

With research questions to guide their choices, 2 researchers working together and constantly comparing their findings can arrive at agreement on the significance of the narrative content and the conclusions that can be drawn from it [18]. The third researcher will validate the findings of the thematic analysis, the fourth researcher will undertake the quantitative analysis, and the team will collaborate to reach consensus on the significance of the findings in relation to triangulation with extant colorectal PRE and PRO data.

### Attribute Coding

The first step of the PROD analysis is to identify and classify the key demographic characteristics of the patient contributors, their disease states, the features of their diagnostic pathways, and the clinical features of their treatment and posttreatment phases. We will organize these data in a framework of yes/no and multiple choice categories. The sets of selections are based on conventional research participant attributes and adapted to the level of detail obtainable from unsolicited narratives. In these accounts, attributes such as age, gender, relationship status, and current disease status information may be unknown from the basic information provided in a source website. These characteristics may only be identifiable with close attention to both content and language in a narrative [52], and even then, may only be inferred from implicit clues [40].

### PRE and PRO Domain Coding

**Figure 3:**
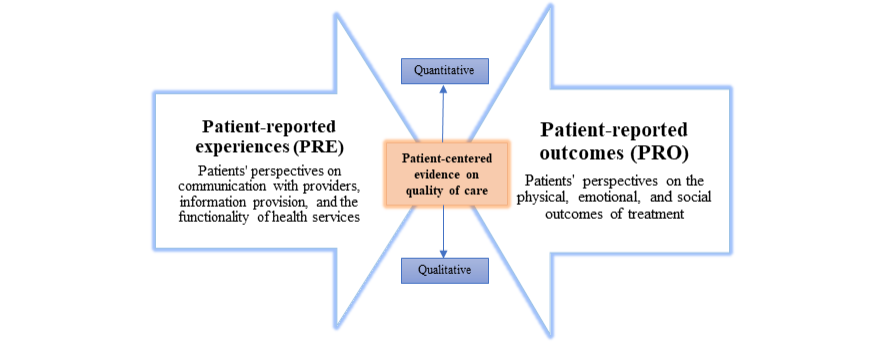
Mixed-methods approach for capturing different dimensions in patient-reported data.

To develop a set of domains and subdomain items for the a priori analytical framework, we reviewed literature on core outcome sets for PRE and PRO measures [2,23,53] and mixed-methods approaches for analyzing PRE and PRO data [12,54-57]. Our conceptual approach to PRE and PRO domain coding is presented in Figure 3. The domains and subdomain items comprised in our analytical framework are presented in Table 1 and also described below.

**Table 1:** Analysis of personal recollections organized as data using the a priori coding framework.

Domains, subdomains					Measures
**Attribute coding**					
	Population characteristics			Gender, marital status, children, date of publication	
	Disease characteristics and management			Age at diagnosis, current status of disease/diagnosis, stage and type of bowel cancer at diagnosis, type of initial medical consultation for symptoms, family history of CRC^a^, investigation for CRC, time from first consultation for illness symptoms to first diagnosis of CRC, discussion of immunotherapy/precision treatment, clinical trials, biomarker-based approach	
	Route to diagnosis			Symptoms prior to first diagnosis, diagnosis received prior to CRC diagnosis, treatments given for diagnosis prior to CRC, investigations undertaken to diagnose CRC, other conditions and genetic syndromes discussed	
	Treatment and posttreatment			Treatment received, posttreatment effects	
**Domain coding**					
	**Patient-reported experience**				
		Functional	Financial impact or costs associated with care Physical context (access, cleanliness, and comfort) Process (continuity and co-ordination of care, scheduling, and waiting times) Quality and efficiency of clinical care		
		Relational	Collaborative nature of interactions (provider and admin) Informational or educational nature of interactions (clinical and practical information, scheduling and waiting times) Interpersonal nature of the interactions (provider and admin)		
	**Patient-reported outcome**				
		Everyday living or usual activities	Caring for family or dependents Domestic chores Gastrointestinal function Getting around or mobility Holidays Independence Living conditions and environment Personal or self-care Recreation		
		Money matters	Finances or financial services Planning the future Work		
		Self and others	Anxiety or depression Body image Existential matters Isolation Pain or discomfort Sexual matters Starting a new family Support and communication		
		Additional issues	Others		

^a^CRC: colorectal cancer.

#### PRO Domains

We reviewed general cancer and colorectal-specific PRO instruments [2,23,53,58,59], including the European Quality of Life Questionnaire-5 dimension (EQ-5D) [60], which assesses health outcomes of care across 5 quality of life domains—anxiety/depression, mobility, pain/discomfort, self-care, and usual activities [60]; the European Organization for Research and Treatment of Cancer-Quality of Life Questionnaire-29-item colon and rectum cancer-site specific (EORTC-QLQ-CR29), the Medical Outcomes Study 12-Item Health Survey, the Functional Assessment of Cancer Therapy-Colorectal (FACT-C), Edmonton Symptom Assessment System, and the Social Difficulties Inventory instrument-21 item (SDI-21), which assesses the impact of cancer on family life, social activities, personal matters, finances, and work. Of these, we selected the SDI-21 and the EQ-5D as being the most relevant to our research interests and for the collection of data from unsolicited free text narratives. We selected these instruments based on the volume of applications in the context of colorectal cancer [12,13,58,61], the applicability of these instruments in people with colorectal cancer across all disease stages and phases of treatment [59,61], and because the domains and items comprised in these instruments offer a balance of broad functional and psychosocial outcomes [13,61-63].

We used 3 core outcome sets from the SDI-21 as the thematic domains for PRO coding: “Everyday Living,” “Money Matters,” and “Self and Others.” We also added a category for “Additional issues” to capture events and perspectives not comprised in these thematic domains. Where possible, we consolidated individual scaled items from SDI-21 outcome sets. For example, in the domain “Money Matters,” we absorbed the items “Welfare benefits,” “Finances,” and “Finance services” into a single item called “Finances or financial services.” Similarly, we synthesized 3 communication and support items into 1 item called “Support and communication.” We also incorporated the SDI-21 single item set into 3 core thematic domains, bringing “Sexual Matters” and “Plans to have a family” into the “Self and others” domain and “Holidays” and “Where you live” into the “Everyday living” domain (Table 1).

While the EQ-5D questionnaire and SDI-21 feature common outcomes, the EQ-5D instrument also accounts for issues relating to pain and discomfort and the psychosocial aspects of everyday life, such as anxiety and depression. We included these items in the framework domain called “Self and others.” To code for issues that are particular to people with colorectal cancer and to cover all items included in colorectal cancer-specific PRO questionnaires such as EORTC-QLQ-CR29 and FACT-C [53], we introduced the item, “Gastrointestinal function” into the “Everyday living” domain.

#### PRE Domains

PRE-questionnaires are commonly designed to examine patients’ experiences of particular health organizations, such as the National Health Service National Cancer Patient Experience Survey, or the services offered in certain health settings [64]. The EORTC, for example, publishes PRE-questionnaires specific to inpatients and inpatient experiences, communication with professionals, and information provision. Given that our data set was drawn from websites in 3 countries and that contributors chose the aspects of their experiences that they wished to describe, we required a broad-ranging generic set of PRE domains and subdomain items for our analytical framework [64].

Rather than selecting domains from a particular PRE instrument, we reviewed the literature to identify the core concepts underpinning PRE-questionnaires. We identified that patient experience outcomes are measured broadly for either relational or functional aspects of experience [64-66]. We used these as the 2 PRE domains in our analytical framework. Relational outcomes account for the interpersonal nature of patient-provider communications, patient-provider collaboration, and information provision to patients [65,67]. Functional outcomes account for the organizational and practical aspects of care, environments of care delivery, and the financial impact of care [65,67] (Table 1).

### Patient Pathway Coding

From patients’ perspectives, experiences of health services and outcomes of care occur as a continuum of patient journey within and across the phases of the patient pathway [40]. We will undertake a patient pathway analysis of the PRE and PRO data by coding for 3 key phases of the patient pathway: diagnosis, treatment, and survivorship/palliative care (Figure 2) [36,68,69].

### Thematic Analysis

There are 4 steps in our thematic analysis: coding for concepts, categorizing codes into groups, detecting patterns across categories, and interpreting themes within and across these patterns [70]. This process transforms the text into a narrative dataset, moving from highly descriptive findings to highly interpretative findings [16,20].

### Quantitative Analysis

We will investigate opportunities to interpret the data quantitatively by means of network analysis [71] and quantitative text-based analysis, which uses automated natural language processing to analyze topics across different documents [12] and can measure sentiments within texts. This method may draw out aspects that contextualize other findings [12,13]. Quantitative approaches to analyzing unstructured text are emerging; however, as yet, there is little consensus on optimal strategies [12,46].

### Methodological Limitations

Our methods will have limitations, including that we will be dealing with text not written for research purposes, not all text will map to our framework, and the data are subjective and will require interpretation. Additionally, data reported in different health systems will need to be seen in the light of those structural and contextual differences. Further, regardless of validity, there are limitations to standardized instruments and these limitations will be reflected in the a priori domains and dimensions that are the foundation of our analytical framework.

## Results

After searching the 3 colorectal cancer patient support and advocacy websites selected for this study, we found that each featured story meets all the inclusion criteria. All texts were downloaded from the internet into the NVivo analysis software, and analysis commenced in September 2020 on the 30 texts randomly selected for this study. We will complete the analysis in March 2021 and we aim to publish the results in mid-2021.

## Discussion

The PROD method for systematically extracting relevant patient-reported data from free-text patient stories aims to maximize the benefits of rich detailed patient-perspective data that can be drawn from patient narratives while framing findings to facilitate data triangulation with patient-reported results from PROs and PREs. Young-onset colorectal cancer is a contemporary issue in need of substantial research input [32,69]. We believe that this will be the first qualitative study to identify and describe the patient pathway from self-symptom identification to help-seeking through diagnosis, treatment, and into survivorship or palliation for people with young-onset colorectal cancer. Unsolicited autobiographical narratives offer a unique opportunity to collect patient-reported data that expose this real-world perspective [40], which is particularly valuable in this age of SARS-COV-2.

The findings from this study have the potential to provide information in a form that can modify habitual thinking and influence clinicians’ cognitive biases [72,73] about age-related criteria for colorectal cancer risk assessment and diagnostic practice. Knowledge of the diagnostic and therapeutic experiences of patients with young-onset colorectal cancer may facilitate greater awareness of colorectal cancer symptoms in people younger than 50 years [74], promote patient proactivity in seeking help, and highlight the importance of identifying hereditary conditions that predispose young people to colorectal cancer [28,75,76]. There is significant potential for the patient-reported data from this study to make a real-world difference to people with young-onset colorectal cancer.

## Abbreviations

EORTC-QLQ-CR29: European Organization for Research and Treatment of Cancer-Quality of Life Questionnaire-29-item colon and rectum cancer-site specific
EQ-5D: European Quality of Life Questionnaire-5 dimension
FACT-C: Functional Assessment of Cancer Therapy-Colorectal
PRE: patient-reported experience
PRO: patient-reported outcome
PROD: personal recollections organized as data
SDI-21: Social Difficulties Inventory instrument-21 item
